# Modeling Survival Time to Death among Stroke Patients at Jimma University Medical Center, Southwest Ethiopia: A Retrospective Cohort Study

**DOI:** 10.1155/2023/1557133

**Published:** 2023-11-29

**Authors:** Bikiltu Wakuma Negasa, Teramaj Wongel Wotale, Mesfin Esayas Lelisho, Legesse Kassa Debusho, Kibrealem Sisay, Wubishet Gezimu

**Affiliations:** ^1^Department of Statistics, College of Natural and Computational Sciences, Mattu University, Mattu, Ethiopia; ^2^Department of Statistics, College of Natural and Computational Sciences, Mizan-Tepi University, Tepi, Ethiopia; ^3^Department of Statistics, University of South Africa, Pretoria, South Africa; ^4^Department of Statistics, College of Natural and Computational Sciences, Jimma University, Jimma, Ethiopia; ^5^Department of Nursing, College of Health Sciences, Mattu University, Mattu, Ethiopia

## Abstract

**Background:**

Stroke is a life-threatening condition that occurs due to impaired blood flow to brain tissues. Every year, about 15 million people worldwide suffer from a stroke, with five million of them suffering from some form of permanent physical disability. Globally, stroke is the second-leading cause of death following ischemic heart disease. It is a public health burden for both developed and developing nations, including Ethiopia.

**Objectives:**

This study is aimed at estimating the time to death among stroke patients at Jimma University Medical Center, Southwest Ethiopia.

**Methods:**

A facility-based retrospective cohort study was conducted among 432 patients. The data were collected from stroke patients under follow-up at Jimma University Medical Center from January 1, 2016, to January 30, 2019. A log-rank test was used to compare the survival experiences of different categories of patients. The Cox proportional hazard model and the accelerated failure time model were used to analyze the survival analysis of stroke patients using R software. An Akaike's information criterion was used to compare the fitted models.

**Results:**

Of the 432 stroke patients followed, 223 (51.6%) experienced the event of death. The median time to death among the patients was 15 days. According to the results of the Weibull accelerated failure time model, the age of patients, atrial fibrillation, alcohol consumption, types of stroke diagnosed, hypertension, and diabetes mellitus were found to be the significant prognostic factors that contribute to shorter survival times among stroke patients.

**Conclusion:**

The Weibull accelerated failure time model better described the time to death of the stroke patients' data set than other distributions used in this study. Patients' age, atrial fibrillation, alcohol consumption, being diagnosed with hemorrhagic types of stroke, having hypertension, and having diabetes mellitus were found to be factors shortening survival time to death for stroke patients. Hence, healthcare professionals need to thoroughly follow the patients who pass risk factors. Moreover, patients need to be educated about lifestyle modifications.

## 1. Introduction

A stroke is a serious illness that is classified as both a cardiovascular and neurologic condition. It is defined as the sudden onset of clinical signs of a focal disturbance in cerebral functions that lasts for 24 hours or longer with no apparent causes other than vascular origin [1–3]. It is a noncommunicable disease (NCD) with significant socioeconomic consequences worldwide [4]. Ischemic and hemorrhagic strokes are the two most common types of stroke, and both are dangerous and life-threatening conditions [5–7]. Every year, about 15 million people worldwide suffer from a stroke, of which around five million suffer from some form of permanent physical disability [8]. Globally, the burden of stroke-related mortality, morbidity, and disability is increasing currently [9–11].

Despite declining rates of incidence, stroke remains the leading cause of death globally [12]. NCDs are more prevalent in developing countries than in the rest of the world [13]. According to the Global Burden of Disease Project, stroke caused 642,000 years of life to be lost due to premature mortality in 2010, a 31% increase since 1990 [14]. In Ethiopia, stroke was the most burdensome NCD and the 13^th^ highest overall cause of years of life lost when compared to other causes of burden [14]. According to a 2012 surveillance study of adult deaths in Addis Ababa, NCDs accounted for 51% of all deaths, with stroke accounting for 11% of all deaths [15, 16]. The overall in-hospital mortality rate for stroke in Ethiopia was 18%.

Previous studies reported different risk factors associated with mortality of stroke patients, like older age [17–19], body temperature greater than 7.1 degrees centigrade, potassium level below 2 mmol/l, and creatinine level > 1.2 mg/dl [16], type of stroke, diabetes, severity of the stroke [18], atrial fibrillation, and lower education status [20] were associated with lower survival of stroke patients. Another study found that removing the feeding gastrostomy tube (FGT) at discharge from the rehabilitation hospital, as well as not aspirating during videofluoroscopic surgery (VSS), was linked to a longer survival time for stroke patients [21].

In the current study, the event of interest was the time to death of stroke patients. Survival analysis, which describes the examination of data from a well-defined time origin to the occurrence of a specific event or endpoint [22–24], is an appropriate statistical tool for this type of data. The Cox regression model was used to model the time to death of stroke patients, which is the most commonly used technique in clinical research.

Although stroke is found to be the major cause of death in Ethiopia, there has been little research on the identification of risk factors using strong statistical models. This study mainly identified survival time and its associated factors among stroke patients. The findings of this study may provide clinicians with more information to aid them in the development of specific treatment plans, prognosis forecasting, and mortality case tracking.

## 2. Methods and Materials

### 2.1. Study Design, Area, and Period

A retrospective study was conducted at the Jimma University Medical Center (JUMC) from January 1, 2016, to January 30, 2019. The JUMC is located about 352 kilometers southwest of Addis Ababa, the capital of Ethiopia. The center serves around 15 million people from the Oromia Regional State and the surrounding regions, including the Southwestern Peoples Regional State and Gambella Regional State. Annually, an estimated total of 12,000, 16,000, 4500, and 220,000 emergency, inpatient, delivery, and outpatient cases attend the center, respectively.

### 2.2. Population Inclusion and Exclusion Criteria

The data were collected from a total of 432 adult patients diagnosed with stroke cases in the medical and surgical wards at JUMC.

**Figure 1:**
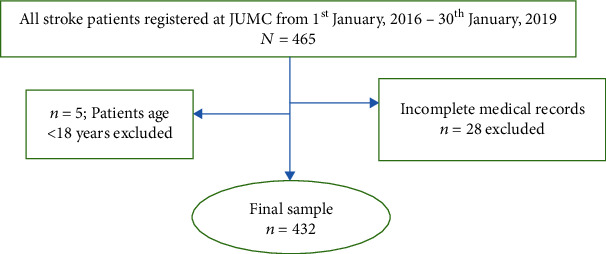
Sampling procedure used to model survival time to death among stroke patients at Jimma University Medical Center, Southwest Ethiopia.

All stroke patients registered with complete information about the study's interest, and patients who took at least one stroke treatment were included in this study. However, patients under 18 years of age, patients who left the study without starting any stroke treatment, and those with incomplete medical records were excluded from the study (Figure 1).

### 2.3. Variable in the Study

The response (outcome) variable in this study was the survival time to death among stroke patients. It was measured in days from the commencement date of stroke treatment to the date of death or censorship. Age, residence, gender, smoking habit, types of stroke, alcohol consumption, hypertension, atrial fibrillation, and diabetes mellitus were variables tested for their influence on the survival time of stroke patients.

### 2.4. Method of Data Analysis

Survival analysis is a group of statistical procedures for data analysis in which the response variable of interest is the time until an event happens. The survivor function is defined as the probability that the survival time of a randomly selected subject is greater than some specified time.

(1) $$\begin{array}{c} S\left(t\right)=P\left(T>t\right)=1-F\left(t\right),t\geq 0, \end{array}$$

which is a nonincreasing function and defined on [0, 1] as *t* ∈ [*o*, ∞]. The hazard function is a measure of the risk of the event happening at any point in time [13]. The hazard function *h* (*t*) ≥ 0 is given as

(2) $$\begin{array}{c} h\left(t\right)=\underset{\Delta t⟶0}{\lim}\frac{t\leq T<t+\left.\Delta t\right|T\geq t}{\Delta t}=\frac{\left(f\left(t\right)\right)}{\left(S\left(t\right)\right)}=-\frac{d}{dt}\;\ln\left[S\left(t\right)\right]. \end{array}$$

Among the other estimators of the survivor function, the Kaplan-Meier estimator is the most common one. The Kaplan-Meier estimator of the survivorship function is also called the product-limit estimator. Kaplan and Meier [14] developed an estimator for the survival function, which is defined as follows:

(3) $$\begin{array}{c} {\hat{s}}_{\left(t\right)}=\underset{t_{\left(j\right)}\leq 1}{\prod }\left(\frac{n_{j}-d_{j}}{n_{j}}\right), \end{array}$$

where *d*_*j*_ is the number of individuals who experienced the event at time *t*_*j*_ and *n*_*j*_ is the number of individuals who have not yet experienced the event at that time. The nonparametric methods are not useful for controlling the covariates, and they require categorical predictors. Therefore, the Cox PH model is a multiple regression method and is used to evaluate the effect of multiple covariates on survival [15]. A broadly applicable and most widely used method of survival analysis, we used partial likelihood estimation: then, *P* (individual has experienced an event at time *t*_*i*_| one event at time *t*_*i*_). When there are no tied times assumed, the partial likelihood is defined over all failure times *t*(*i*), where *i* = 1, 2, ⋯*m*, and given as

(4) $$\begin{array}{c} L\left(\beta \right)=\overset{m}{\underset{i=1\;}{\prod }}\frac{\exp\left(\beta^{\prime }x_{i}\right)}{\sum_{j\;\epsilon \;R_{i\left(j\right)}}\exp\left(\beta^{\prime }x_{j}\right)}. \end{array}$$

In the statistical area of survival analysis, an accelerated failure time (AFT) model is a parametric model that can be used as an alternative to the PH model, especially to overcome the statistical problems due to the violation of the PH assumption [16]. The survival function for a group of patients with covariates (*x*_1_, *x*_2_,*…*, *x*_*p*_) can be expressed as

(5) $$\begin{array}{c} S\left(t\right)=S_{0}\left(\phi t\right), \end{array}$$

where *S*_0_(*t*) is the baseline survival function and *ϕ* is an acceleration factor defined to be

(6) $$\begin{array}{c} \phi =\exp\left\{\left(\alpha_{1}x_{1}+\alpha_{2}x_{2}+\cdots +\alpha_{p}x_{p}\right)\right\}. \end{array}$$

The log-linear form of the AFT model for survival time assumes that the relationship between the logarithm of survival time*T*_*i*_, associated with the lifetime of the *i*^th^ individual in a study, and the corresponding covariates is linear and can be written as follows:

(7) $$\begin{array}{c} \text{Log}T_{i}=\mu +\alpha_{1}x_{1i}+\alpha_{2}x_{2i}+\cdots +\alpha_{p}x_{pi}+\sigma \epsilon_{i,} \end{array}$$

where Log *T*_*i*_ is the log-transformed survival time, *α*_1_, *α*_2_, ⋯, *α*_*p*_ are the unknown coefficients of the values of *p* explanatory variables *x*_1,_ *x*_2,_ ⋯ *x*_*p*_. The AFT models considered in this study are the exponential, Weibull, and log-normal AFT models to measure the effect of covariates on survival time instead of hazard rate. The AFT models are named for the distribution of *T* rather than the distribution of log*T*.

### 2.5. Model Selection and Diagnostics

One of the most commonly used model selection criteria is the Akaike information criterion (AIC) [17, 18]. The Akaike information criterion (AIC) is a prediction error estimator that determines the relative quality of statistical models for a given set of data. In other words, AIC measures the quality of each model in relation to the other models given a set of data. As a result, AIC provides a method for model selection.

In practice, we begin with a set of candidate models and then calculate the AIC values for each model. Using a candidate model to represent the “actual model,” i.e., the process that created the data, will nearly always result in information loss. We want to choose the model that minimizes information loss from among the candidate models. Hence, given a set of candidate models for the data, the preferred model is the one with the lowest AIC value.

For comparing nonnested models, Akaike's information criterion (AIC), which is defined as

(8) $$\begin{array}{c} \text{AIC}=-2\text{L}+2k, \end{array}$$

where *L* is the log-likelihood and *k* is the number of estimated parameters in the model.

After a model is fitted, its adequacy needs to be assessed. The methods involved in this study are checking the adequacy of the parametric baselines and the Cox-Snell residuals [19].

## 3. Result and Discussion

### 3.1. The Descriptive Summaries

Of the 432 patients who followed the study, 223 (51.6%) experienced the event of death, and the remaining 209 (48.4%) were censored. The overall median death time from stroke was 15 days. The minimum and maximum follow-up times were 1 and 69 days, respectively. About 236 (54.6%) of the patients were female, of whom about 114 (48.3%) and 122 (51.7%) were dead and censored, respectively. Among the total number of stroke patients, 196 (45.4%) were males, of whom 109 (55.6%) and 87 (44.4%) were dead and censored, respectively. The median death times of male and female patients were 13 and 16 days, respectively (Table 1).

#### 3.1.1. Kaplan-Meier Survivor Estimates for Some Covariates of Stroke Patients

**Figure 2:**
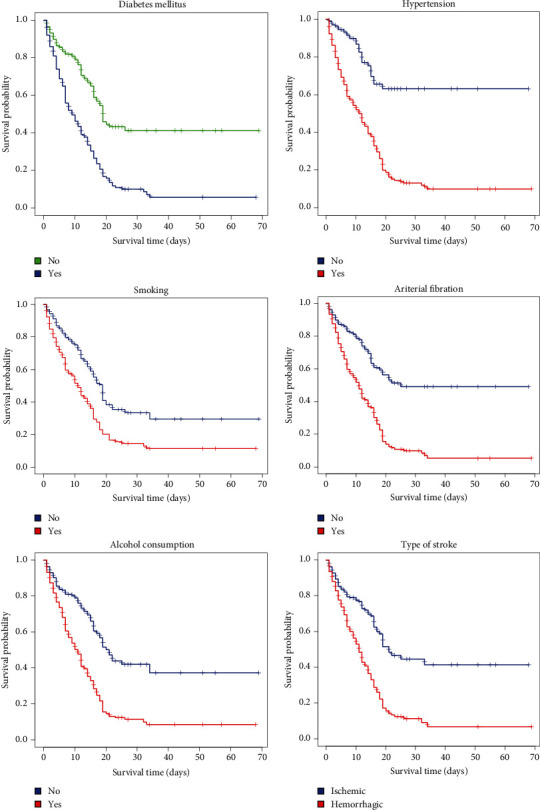
The Kaplan-Meier survivor estimates for some covariates of stroke patients at Jimma University Medical Center, Southwest Ethiopia.

To compare the survival time of individual patients with each level of categorical covariates, the Kaplan-Meier method and log-rank test were applied. The Kaplan-Meier survivor estimates for alcohol consumption showed that patients who did not drink alcohol had a longer survival time than those who did. Comparing the survivor functions between smoker and nonsmoker stroke patients, the Kaplan-Meier showed that nonsmoking patients had higher survival times than smokers. Patients with an ischemic stroke had a longer survival time than patients who had a hemorrhagic stroke. The Kaplan-Meier survivor estimates also showed that patients who had no hypertension, no diabetes mellitus, and no atrial fibrillation had a higher survival time than their counterparts (Figure 2).

#### 3.1.2. Test of Proportional Hazard Assumption

**Figure 3:**
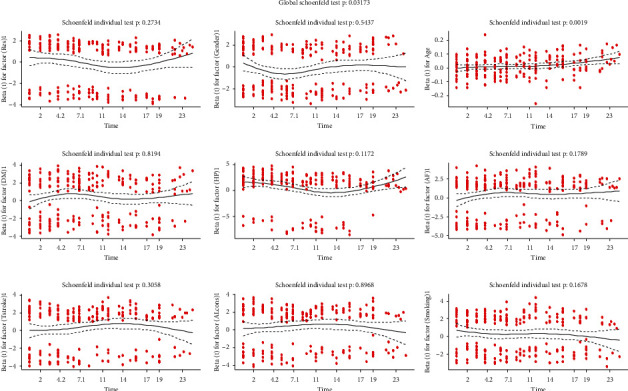
The Schoenfeld residuals for some selected covariates of stroke patients at Jimma University Medical Center, Southwest Ethiopia.

The proportionality of the Cox proportional hazard model can be tested using the rho statistic, *p* value, and scaled Schoenfeld residuals. The large value of rho showed a strong correlation between residuals and time; because of this, there is a systematic pattern on the graph that shows that the proportional hazard assumption is not satisfied. The *p* value of the rho statistic in Table 2 is less than 5% for the given covariates, indicating the rejection of the null hypothesis of the proportionality of the Cox proportional hazard model. Because of this, the *p* value of the covariate age was less than 5%. Also, it is revealed that the plot of scaled Schoenfeld residuals against transformed time indicates a systematic departure from a horizontal line (Figure 3), so the covariate does not fulfill the proportional hazard assumption. For the global test, the assumptions of Cox PH also failed due to the significance of the result, and the Cox proportional hazard model cannot be used for this data.

#### 3.1.3. Schoenfeld Residuals of Stroke Patients for Some Selected Covariates

The accelerated failure time (AFT) model is another alternative to the Cox PH model when assumptions in the Cox PH are violated. In this study, the data set was fitted by AFT models using different baseline distributions, i.e., Weibull, exponential, and log-normal. To investigate the effect of the candidate covariates on the survival time of stroke patients, we did the first univariable analysis fitted for every covariate by AFT models using different baseline distributions. The multivariable analysis of AFT models in the study was done by assuming the exponential, Weibull, and log-normal distributions for the baseline hazard function. It was performed by using the nine most significant covariates. From the univariable analysis, we observed that the covariates age of the patients, hypertension, atrial fibrillation, diabetes mellitus, types of stroke disease diagnosed, smoking habit, alcohol consumption, gender, and place of residence were significant in the entire Weibull, exponential, and log-normal AFT models. However, we excluded the aim of radiotherapy, the treatment patients received, and the number of children, which were not significant in the univariable analysis.

For the data on stroke patients, a multivariable analysis of AFT models of exponential, Weibull, and log-normal distributions was fitted by including all the covariates that were significant in the univariable analysis. To compare the efficiency of different models, the AIC was used. It is the most common and applicable criterion for selecting a model. Based on AIC, a model with a minimum value of AIC was preferred. Accordingly, from Table 3, the AIC value of the Weibull AFT model (AIC = 1663.329) was the minimum of all the other AIC values of the alternative models, which indicates that it is the most efficient model to describe the stroke data set among the different distributions of AFT models. Hence, the Weibull AFT model was selected as the best fit for the stroke data set.

The final results for the Weibull AFT model are shown in Table 4, and to decide whether or not a variable is significant, the *p* value associated with each parameter has been estimated, and variables that have a *p* value less than or equal to 5% were considered important variables and were included in the study. The survival time of stroke patients was significantly related to their age, atrial fibrillation, alcohol consumption, types of stroke disease diagnosed, hypertension, and diabetes mellitus. This indicates that they are important prognostic factors for the survival time to death of stroke patients. An acceleration factor greater than 1 specifies prolonging the survival time of the event, and an acceleration factor less than 1 indicates shortening the survival time of the event.

The acceleration factor for the age of patients estimated to be 0.98 indicated that, as the age of patients increased by a year, the survival time to death of stroke patients decreased by a factor of 0.98 (=0.98, 95% CI: 0.97–0.99), holding the remaining covariates constant. The survival time to death of patients who had atrial fibrillation was shortened by a factor of 0.67 (=0.67, 95% CI: 0.51–0.88) by keeping the remaining covariates constant.

An estimated acceleration factor for patients consuming alcohol was 0.72, indicating that the survival time to death of stroke patients decelerated by a factor of 0.72 (=0.72, 95% CI: 0.56-0.94) as compared to nondrinkers. The estimated acceleration factor for patients who had the hemorrhagic type of stroke disease was 0.73, indicating that the survival time to death of stroke patients was shortened by a factor of 0.73 (=0.73, 95% CI: 0.55-0.93) by keeping the remaining covariates constant. When we looked at the history of hypertension in stroke patients, the estimated acceleration factor was estimated to be 0.52, which implies that the survival time of the patients was decreased by a factor of 0.52 = 0.52, 95% CI: 0.37–0.75, by holding the other covariates constant.

Finally, observing the history of diabetes mellitus was also known to be a significant covariate. The estimated acceleration factor of patients who had diabetes mellitus was estimated to be 0.68, indicating that the survival time to death of patients decreased by a factor of 0.68 (=0.68, 95% CI: 0.52, 0.88) by keeping the remaining covariates constant.

**Figure 4:**
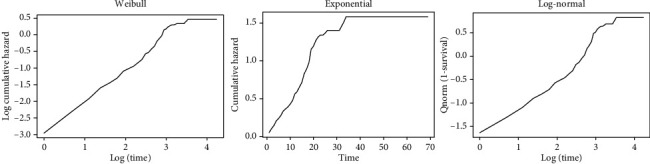
Graphical evaluation of the Weibull, exponential, and log-normal assumptions.

The final step in the model assessment is to see the overall goodness of fit. Therefore, it is desirable to determine whether a fitted parametric model adequately describes the data or not. To check the adequacy of our baseline hazard, the exponential is plotted by −log (*S*(*t*)) with the time of the study; the Weibull is plotted by log(log(*S*(*t*))) with the logarithm of the time of the study; the log-normal is plotted by the q-norm (1 - survival) or *t*) with the logarithm of the time of the study; and the log-normal is plotted by the q-norm (1 - survival) or 1 (1*S*(*t*)) with the logarithm of time (Figure 3). The plot of the Weibull distribution is approximately linear compared to the other plots. The patterns suggest that the Weibull distribution is appropriate for the model (Figure 4).

**Figure 5:**
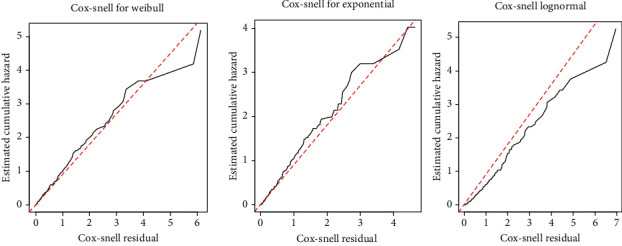
The Cox-Snell residuals obtained by fitting the Weibull, exponential, and log-normal models to the stroke data set.

*(1) Graphical Evaluation of Weibull, Exponential, and Log-Normal Assumptions*. The Cox-Snell residuals are one way to investigate how well the model fits the data. In this case, we used the Cox-Snell residuals to check the overall goodness of fit for different parametric models. By observing the Cox-Snell residuals plot from Figure 5, the Weibull AFT model fits stroke patients' data since the plot of Cox-Snell residuals against cumulative hazard function of residuals is approximately a straight line through the origin with 45 degrees, compared with exponential and log-normal. This indicates that the Weibull AFT model is an adequate, efficient, and appropriate model for analyzing stroke patients' data at Jimma University Medical Center.

## 4. Discussion

This study is aimed at identifying factors affecting the survival time of stroke patients using a data set obtained from Jimma University Medical Center. The univariable and multivariable analyses of survival models were employed to examine the factors that affect the time to death of stroke patients. A Cox PH model was applied to this study. However, when using the Cox PH model, the proportional hazard assumption needs to be satisfied to make trustworthy inferences from a particular data set. But its assumptions were violated for some reason based on the nature of the data set.

Although taking the covariate associated with violence in the PH assumption as a stratification variable for categorical covariates and adding time-dependent covariates are solutions, in such cases, the AFT model is more informative and the best alternative to use. Hence, the variable age did not fulfill the Cox proportional hazard assumption for this data set. Therefore, the AFT models with baseline distributions (exponential, Weibull, and log-normal) were used. Comparisons of survival models under different distributions of the hazard function provide the best model for fitting the specific data with appropriate inference [20]. In this study, the Weibull AFT model has the smallest AIC, indicating its ability to fit the data.

More than half (51.6%) of patients experienced the event of death in this study. From the multivariable analysis of the Weibull accelerated failure time model, the survival time (time to death) of stroke patients was significantly affected by age, hypertension, atrial fibrillation, diabetes mellitus, alcohol consumption, and the type of stroke disease diagnosed. The age of the patient was one of the risk factors that affected the survival time to death of stroke patients. This means that older patients had a higher hazard rate. This finding was similar to an earlier study conducted in Ethiopia [21, 22], in the northwest of Iran [23], and in Australia and New Zealand [24]. This might be due to the elderly patients' increased risk of severe stroke complications, which further decreases the patients' survival time.

The hazard for patients with atrial fibrillation was higher compared with patients who did not have atrial fibrillation. These results also coincide with the study done in Ethiopia [25, 26]. This implies that atrial fibrillation is a heart condition that causes an irregular and often abnormally fast heartbeat, and atrial fibrillation has a significant effect on increasing the risk of stroke. The findings of this study revealed that alcohol consumption had a significant effect on the time to death of stroke patients. These results are consistent with findings from a study in Ethiopia [27, 28]. This might be due to the regular consumption of large amounts of alcohol, which greatly increases the risk of having a stroke [29], as it can lead to high blood pressure, diabetes, obesity, and atrial fibrillation. Drinking too much alcohol can also damage the liver and stop it from making substances that help our blood clot, increasing our risk of having a stroke caused by a bleed. In this study, hypertension was identified as a significant factor that influences the survival time to death of stroke patients. According to these studies, hypertension is consistently and independently associated with the risk of mortality from stroke and has a higher hazard rate than patients without hypertension. Hence, this result is similar to other findings obtained by [25, 28, 30]. This is due to the fact that hypertension means that the blood is exerting more pressure than is normal or healthy. Over time, this weakens and damages the blood vessel walls and thickens the artery walls, resulting in narrowing and eventual blockage of the vessel, which can lead to stroke. The findings of this study showed that diabetes mellitus was identified as a significant risk factor for the time to death of stroke patients. The finding is confirmed by a previous study [31, 32]. This indicates that patients who had no diabetes mellitus had a higher survival probability than patients who had diabetes mellitus. This might be due to the fact that diabetes is a chronic condition in which the body is unable to utilize blood sugar. This is because high blood sugar levels contribute to the development of atherosclerosis (narrowing of the arteries), which reduces survival time. In addition to those variables, the type of stroke disease diagnosed also has a significant effect on the time from diagnosis to death for stroke patients. The finding illustrates that the risk of death due to the hemorrhagic type of stroke disease is higher than for patients who had the ischemic type of stroke. This study was justified by a study conducted in Ethiopia [33] and Japan [34], showing that the survival time for patients who had the hemorrhagic type of stroke disease was shorter than ischemic. This is due to the fact that hemorrhagic stroke is extremely dangerous because the blood in the brain can sometimes lead to further complications such as hydrocephalus, increased intracranial pressure, and blood vessel spasms, which can lead to severe brain damage and even death.

### 4.1. Limitation of the Study

The study employed a strong statistical model to predict time to death among stroke patients. However, since it utilized secondary data, some important risk factors for stroke, like physical inactivity, family history of stroke, and obesity, were not tested in this study because these covariates were not completely documented in the patient's record.

## 5. Conclusion and Recommendation

According to the results of the Weibull accelerated failure time model, older age, having atrial fibrillation, alcohol consumption, being diagnosed with hemorrhagic types of stroke disease, having hypertension, and having diabetes mellitus significantly shortened the time to death of stroke patients. Special attention should be paid to those patients based on this significant variable to improve their health.

### 5.1. Implications for Clinical Practices

Healthcare professionals need to promptly assess and recognize the patient's conditions that can affect a stroke prognosis, including older age, atrial fibrillation, alcohol consumption, hemorrhagic types of stroke, hypertension, and diabetes mellitus. Accordingly, they need to educate their patients about risk factors and lifestyle modifications to halt stroke-related death. Moreover, close attention should be given to old patients and those who have atrial fibrillation and hemorrhagic strokes.

## Figures and Tables

**Table 1:** Descriptive summary of stroke patients (2016-2019).

Variable	Category	No. of patients	Status of patients		Median (days)
			Censoring	Event	
Gender	Male	196	87 (44.4%)	109 (55.6%)	13
	Female	236	122 (51.7%)	114 (48.3%)	16

Residence	Urban	162	90 (55.6%)	72 (44.4%)	16
	Rural	270	119 (44.1%)	151 (55.9%)	15

Alcohol consumption	No	241	163 (67.6%)	78 (32.4%)	21
	Yes	191	46 (24.1%)	145 (75.9%)	11

Smoking habit	No	253	152 (60.1%)	101 (39.9%)	19
	Yes	179	122 (68.2%)	57 (31.8%)	11

Types of stroke diagnosed	Ischemic	233	159 (68.2%)	74 (31.8%)	21
	Hemorrhagic	199	50 (25.1%)	149 (74.9%)	11

Hypertension	No	172	139 (80.8%)	33 (19.2%)	28
	Yes	260	70 (26.9%)	190 (73.1%)	11

Atrial fibrillation	No	232	167 (72%)	65 (28%)	25
	Yes	200	42 (21%)	158 (79%)	11

Diabetes mellitus	No	268	181 (67.5%)	87 (32.5%)	19
	Yes	164	28 (17.1%)	136 (82.9%)	9

Total		432	209 (48.4%)	223 (51.6%)	15

**Table 2:** Test of proportional hazard assumption.

Covariates	Rho	Chi-square	*p* value
Gender	0.040	0.369	0.543
Age	0.217	9.676	0.001
History of DM	0.014	0.052	0.819
History of HP	-0.097	-0.097	0.117
Residence	-0.072	1.199	0.273
Atrial fabrication	0.083	1.807	0.178
Smoking	-0.090	1.902	0.167
Alcohol use	0.008	0.017	0.896
Types of stroke diagnosed	0.062	1.048	0.305
Global		18.311	0.031

**Table 3:** Comparisons of AFT models using AIC.

Baseline distribution	Exponential	Weibull	Log-normal
AIC value	1667.169	1663.329	1671.616

**Table 4:** Result of multivariable Weibull AFT model.

Covariates	Categories	Coef	SE	*ϕ*	95% CI for *ϕ*	*p* value
Age	Continuous	-0.01	0.004	0.98	(0.97, 0.99)	<0.001^∗∗^
Atrial fibrillation (ref: no)	Yes	-0.39	0.14	0.67	(0.51, 0.94)	0.004^∗∗^
Alcohol consumption (ref: no)	Yes	-0.32	0.13	0.72	(0.56, 0.94)	0.013^∗^
Types of stroke (ref: ischemic)	Hemorrhagic	-0.33	0.13	0.73	(0.55, 0.93)	0.011^∗^
Hypertension (ref: no)	Yes	-0.65	0.18	0.52	(0.37, 0.75)	<0.001^∗∗^
Diabetes mellitus (ref: no)	Yes	-0.38	0.13	0.68	(0.52, 0.88)	0.003^∗∗^

Coef = estimated value of coefficients; SE = standard error, *ϕ* = acceleration factor; 95% CI = 95% confidence interval for acceleration factor; ref = reference. ^∗^Statistically significant at 5% level.

## Data Availability

The data used to support the findings of this study are available from the corresponding author upon request.

## Abbreviations

AFT: Accelerated failure time
AIC: Akaike's information criterion
CI: Confidence interval
JUMC: Jimma University Medical Center
HR: Hazard ratio
NCD: Noncommunicable disease
PH: Proportional hazard.
